# Neural Networks for Modeling Neural Spiking in S1 Cortex

**DOI:** 10.3389/fnsys.2019.00013

**Published:** 2019-03-29

**Authors:** Alice Lucas, Tucker Tomlinson, Neda Rohani, Raeed Chowdhury, Sara A. Solla, Aggelos K. Katsaggelos, Lee E. Miller

**Affiliations:** ^1^Department of Electrical Engineering and Computer Science, Northwestern University, Evanston, IL, United States; ^2^Department of Physiology, Northwestern University, Chicago, IL, United States; ^3^Department of Biomedical Engineering, Northwestern University, Evanston, IL, United States; ^4^Department of Physics and Astronomy, Northwestern University, Evanston, IL, United States; ^5^Department of Physical Medicine and Rehabilitation, Northwestern University and Rehabilitation Institute of Chicago, Chicago, IL, United States

**Keywords:** somatosensory cortex, limb-state encoding, single neurons, reaching, monkey, artificial neural networks

## Abstract

Somatosensation is composed of two distinct modalities: touch, arising from sensors in the skin, and proprioception, resulting primarily from sensors in the muscles, combined with these same cutaneous sensors. In contrast to the wealth of information about touch, we know quite less about the nature of the signals giving rise to proprioception at the cortical level. Likewise, while there is considerable interest in developing encoding models of touch-related neurons for application to brain machine interfaces, much less emphasis has been placed on an analogous proprioceptive interface. Here we investigate the use of Artificial Neural Networks (ANNs) to model the relationship between the firing rates of single neurons in area 2, a largely proprioceptive region of somatosensory cortex (S1) and several types of kinematic variables related to arm movement. To gain a better understanding of how these kinematic variables interact to create the proprioceptive responses recorded in our datasets, we train ANNs under different conditions, each involving a different set of input and output variables. We explore the kinematic variables that provide the best network performance, and find that the addition of information about joint angles and/or muscle lengths significantly improves the prediction of neural firing rates. Our results thus provide new insight regarding the complex representations of the limb motion in S1: that the firing rates of neurons in area 2 may be more closely related to the activity of peripheral sensors than it is to extrinsic hand position. In addition, we conduct numerical experiments to determine the sensitivity of ANN models to various choices of training design and hyper-parameters. Our results provide a baseline and new tools for future research that utilizes machine learning to better describe and understand the activity of neurons in S1.

## Introduction

“Encoding" models, which describe neural firing as a function of externally observed variables, have widespread utility in both basic neuroscience and neural engineering. The encoded variables typically are measures of limb state, such as hand position or joint angles. Modeling these relations serves as an engineering tool for quantifying a mapping between neural state and limb state, and can serve as the foundation for afferent brain machine interfaces (Bensmaia and Miller, 2014; Saal et al., 2017). It can also illuminate the way that neurons encode information within the nervous system. Comparisons between models relating neural activity to extrinsic representations of limb state such as hand position, and those which use intrinsic representations of limb state such as joint angles or muscle length, can provide insight into the features of posture and movement encoded by neurons at different CNS levels.

Here we focus on proprioception, the sense of limb position and movement that is critically important in the control of limb movement; patients who have lost proprioception have great difficulty making precise, coordinated movements (Sainburg et al., 1993, 1995). Proprioception derives from sensors in the muscles, tendons, joints, and skin, with each sensor type responding to different aspects of the mechanics of limb motion, including both kinematics and kinetics. These sensors project information differentially into the four areas (1, 2, 3a, and 3b) of the primary somatosensory cortex (S1) (Friedman and Jones, 1981; Pons et al., 1985; Krubitzer and Kaas, 1990; London and Miller, 2013). Area 2 is the first area in which signals from all these receptor types converge on single neurons. This convergence suggests that activity of neurons in this area may reflect a transformation of the afferent information into a common code, perhaps encoding task relevant extrinsic features such as hand position (Soechting and Flanders, 1989; McIntyre et al., 1997; Lacquaniti and Caminiti, 1998; Cohen and Andersen, 2002). However, these prior works are limited by their exclusive reliance on few, low dimensional, extrinsic signals such as Cartesian hand position, only distantly related to the signals produced by the afferent receptors.

Alternatively, neural activity in area 2 may instead represent lower-level features of limb state, such as change in muscle length or joint angles. If so, correlations with higher-level features such as hand position might simply arise from the relation between intrinsic and extrinsic coordinate systems. If area 2 somatosensory neurons respond to low-level features of the proprioceptive input, variables such as muscle length might provide superior prediction of neural activity than hand position. This is the fundamental issue we seek to address.

In recent years there has been great interest in using machine learning techniques to capture maps between neural state and limb state (Benjamin et al., 2018). Advances in the application of Artificial Neural Networks (ANNs) to neural data have produced promising results in classification, detection, and prediction for various tasks (Goodfellow et al., 2016). The main advantage of using ANNs over other techniques such as Wiener (Pohlmeyer et al., 2007) or Kalman filters (Wu et al., 2003) is that they are more effective in learning intricate structures in the data (Glaser et al., 2017) and in approximating complex nonlinear functions. Recurrent Neural Networks (RNNs) have been employed for modeling motor cortical data for decoding applications where they have substantially outperformed linear methods (Sussillo et al., 2012; Glaser et al., 2017; Han et al., 2017). For proprioceptive neurons, current models are largely limited to simple linear mappings from planar hand position or interaction forces to the firing rate of single cells (Prud’homme and Kalaska, 1994).

In this paper, we use ANNs to model the firing rates of neurons in area 2. Our group has recently developed techniques that allow us to record the full seven degrees of freedom (DOF) configuration of the arms of monkeys as they reach, simultaneously collecting the spiking activity from about 100 neurons. We use this rich dataset to investigate the ability of ANNs to predict the recorded neural activity and to identify the most relevant input features. We explore the kinematic features that provide the best network performance, and find that intrinsic parameters, like muscle length, are more informative than hand kinematics; this suggests that the firing rates of neurons in area 2 may be more closely related to the activity of peripheral sensors than to extrinsic hand position.

While there is great promise in applying machine learning methods to neural encoding and decoding problems, there are also great challenges. Unlike linear methods, the complex and nonlinear nature of ANNs implies no guarantee that a network will learn the appropriate function (Goodfellow et al., 2016). Selection of datasets, data preprocessing, network architecture design, and choice of training hyper-parameters may all affect the performance of the resulting network. Because an exhaustive search over the space of these options is computationally intractable, modelers resort to a trial and error approach, based on past experience and focused on a limited range of choices. Since poor design choices may result in a subpar performance that negates the potential benefits of the ANN approach, we investigate how these training parameters affect the ability of an ANN to predict neural activity. We experiment with various choices of hyper-parameters and training protocols, such as the amount of training data provided to the neural networks and the choice of regularization during training. We also explore the advantages of recurrence over the feature extraction approach of feedforward network design.

We emphasize that the ANNs are not being used here as models of the actual brain networks that connect limb receptors and cortical neurons, but as tools for implementing a map from potentially relevant inputs to neural activity in S1. The comparative analysis of the performance of networks trained on different subsets of inputs is then used to gain novel insights on the encoding properties of S1 neurons. In addition to its specific neuroscience interest, we use this case study to highlight the usefulness of ANN models while emphasizing that their implementation is not automated. There are many decisions to be made when implementing these methods; we provide information on which are the most important and what the implications of the various choices are, so as to guide the selection of appropriate parameters for the application of such machine learning approaches to the analysis of systems neuroscience data.

## Materials and Methods

### Ethics Statement

All animal care, surgical, and experimental procedures in this study are consistent with the Guide for the Care and Use of Laboratory Animals and were approved by the Institutional Animal Care and Use Committee of Northwestern University. Nonhuman primates are an important experimental model in the investigation of motor control and the somatosensory system. The somatosensory and motor areas of the nervous system, as well as the musculoskeletal system of these animals are similar to humans, providing a good analog for the human model. Macaque monkeys are not endangered and are commonly used in laboratories studying motor control and brain machine interfaces, allowing for ready comparison of results across experiments. We take great care that these animals are comfortable and remain in good health, both for humane reasons and because animals that are stressed or in poor health do not perform as well as healthy animals.

### Data Collection

To collect the datasets, three male rhesus macaque monkeys were trained to perform simple visually guided reaching tasks. The monkeys gripped the handle of a robotic manipulandum, and moved the handle within a plane to direct a cursor displayed on a video monitor. The monitor displayed a sequence of randomly placed targets; the monkeys were given a small liquid reward after successfully moving the cursor through a short sequence of targets.

After training, we surgically implanted 96-channel arrays of recording electrodes (Blackrock Microsystems) into area 2. Monkeys were anesthetized with isoflurane gas and a craniotomy was performed above S1. We reflected the dura and isolated the arm area of S1 by recording from the surface of cortex with bipolar silver electrodes while palpating the arm to drive a neural response. Once we located the portion of cortex responding to arm stimulation, we inserted the recording array adjacent to the post-central sulcus. We then replaced the dura and the original bone flap to close the craniotomy, and sutured the skin closed. After surgery, monkeys were provided antibiotics and analgesics to prevent infection and pain.

Once the monkeys had recovered from surgery, we recorded neural data during task performance. Data were bandpass filtered between 250 and 7,500 Hz, and spiking of neurons was collected as a waveform snippet surrounding each threshold crossings of the extracellular voltage. After data collection, the snippets were manually sorted using Offline Sorter (Plexon) to isolate single neurons from background activity. Sorted units were then imported into MATLAB, and spike times were grouped into 50 ms bins to provide a 20 Hz estimate of the firing rate.

To record the configuration of the monkey’s arm, we used custom motion tracking software based on the Microsoft Kinect. We shaved the monkey’s arm and applied paint markers to the skin. Our software computed the three-dimensional position of these markers. Offline, these data were imported into OpenSim (Delp et al., 2007) and the marker locations were registered to a seven DOF model of the monkey’s arm (Chan and Moran, 2006). Using the 7DOF model, we computed the time-varying joint angles and muscle lengths. These data were also resampled at 20 Hz. We modeled one dataset from each monkey, with 60 neurons (length 49 min), 79 neurons (11 min), and 14 neurons (33 min), respectively, and referred to as Datasets H, C, and L.

### Network Training and Performance Measure

To model the mapping from the kinematic variables to the neural firing rates, we used a fully connected feedforward neural network architecture. The number *K* of input units equaled the number of kinematic variables chosen as predictors. The network then consisted of two hidden layers, each composed of 64 units, followed by an output layer with as many units as the number *N* of recorded neurons whose activity the network was trained to predict. Units in the hidden layer used a rectified-linear activation function (Nair and Hinton, 2010) as the static nonlinearity; units in the output layer used an exponential nonlinearity. To establish a baseline for performance comparison, we also trained a Generalized Linear Model (GLM), equivalent to a network without hidden layers, which finds a linear map from *K* input units to *N* exponential output units. Supplementary Figure S1 shows a number of examples of the performance advantage of a multi-layer neural network compared to a GLM, which illuminates the intrinsic nonlinearity of the input-output map captured by the network models.

The design decisions that led to the layered network architecture described above resulted from exploring the ability of the network to predict the activity of the recorded S1 neurons. We explored decreasing and increasing the number of hidden layers by one, and increasing and decreasing the number of hidden units in each layer. We also explored using a hyperbolic tangent nonlinearity for the hidden units. The architecture described above provided the best performance among the various alternatives we explored.

We implemented the networks using the Keras library with Tensorflow v1.1 as a backend and a Titan X GPU card. We trained the networks using the Adam optimizer (Kinga and Adam, 2015), commonly used in the deep learning literature. We also explored using the SGD optimizer (Bottou et al., 2018), but with less successful results.

Given a *K* × 1 input vector ***x**_m_* at time step *m*, the neural network learns a function *f_θ_*(⋅) which maps the *K* input variables to the *N* × 1 vector of binned spike rates ***ŷ**_m_* for all *N* neurons, i.e., ***ŷ**_m_* = *f_θ_*(***x**_m_*) at time step *m*. To solve for the unknown parameters *θ* of the neural network, we considered the likelihood of the data given the model under the Poisson assumption of independent time bins and under the assumption of statistical independence of neural activity conditioned on the inputs, namely:

(1) $$\text{Prob }\left(\left\{y_{\mathrm{nm}}\right\}|\theta \right)=\prod_{n=1}^{N}\prod_{m=1}^{M}\frac{{\left({\hat{y}}_{\mathrm{nm}}\right)}^{y_{\mathrm{nm}}}}{y_{\mathrm{nm}}!}e^{-{\hat{y}}_{\mathrm{nm}}}$$

Here *ŷ_nm_* is the predicted firing rate of the *n*-th neuron at time bin *m* (the *n*-th element of the vector ***ŷ**_m_*), and *y_nm_* is the measured firing rate of the *n*-th neuron at time bin *m*. The Poisson loss function to be minimized is minus the logarithm of the likelihood of the data given the model, which is normalized by the number *N* of predicted neurons and the number *M* of time bins (the number of samples in the dataset) to obtain:

(2) $$L=\frac{1}{\mathrm{NM}}\sum_{n=1}^{N}\sum_{m=1}^{M}\left({\hat{y}}_{\mathrm{nm}}-y_{\mathrm{nm}}\log\left({\hat{y}}_{\mathrm{nm}}\right)\right)$$

In this approach, instead of training a separate model for each neuron *n*, we trained a single neural network to predict the activity of all neurons simultaneously. Therefore, for each time bin *m*, the network takes a *K* × 1 vector ***x**_m_* as its input and returns an *N* × 1 output vector ***ŷ**_m_* of the predicted firing rates of all neurons at that time. It is implicitly through these outputs that the loss function depends on the parameters *θ*. As has been established in many years of accumulated experience training feedforward neural networks, a training approach such as this one, based on fitting the firing activity of all neurons simultaneously, allows the network to develop richer task-appropriate internal representations, which in this case capture information about the population firing rate covariance as well as individual firing rates.

We trained the network by gradient descent using batch sizes of 128 data points to perform our weight updates. We define one epoch as the number of iterations necessary for the weight updates to incorporate changes due to all data points in the training set. We set the maximum number of training epochs to 200, but training stopped earlier due to the use of an early stopping procedure for regularization. A loss was computed at the end of each epoch, as the average loss over a validation dataset that comprised a randomly selected 20% of the training data. The early stopping procedure halted the training when the validation loss computed at the end of one epoch exceeded its value at the preceding epoch. This procedure typically stopped the training before 50 epochs.

The performance of the trained networks was evaluated using a 10-fold cross validation procedure within each of the three datasets. To implement cross validation, the data was randomly divided into 10 subsets, the folds. In each experiment, one fold was used as the test dataset, and the others were used for training/validation. In all figures we report mean ± standard deviation across the folds. Example learning curves, one for each of the three datasets, are provided in Supplementary Figure S2.

We quantify the performance of the trained neural network with the pseudo-R^2^ measure (*pR*^2^), denoted for each neuron *n* as ${\mathrm{pR}}_{n}^{2}$, and defined as:

(3) $${\mathrm{pR}}_{n}^{2}=1-\frac{\sum_{m=1}^{M}(y_{\mathrm{nm}}\log\left(y_{\mathrm{nm}}\right)-y_{\mathrm{nm}})-\sum_{m=1}^{M}\left(y_{\mathrm{nm}}\log\left({\hat{y}}_{\mathrm{nm}}\right)-{\hat{y}}_{\mathrm{nm}}\right)}{\sum_{m=1}^{M}(y_{\mathrm{nm}}\log\left(y_{\mathrm{nm}}\right)-y_{\mathrm{nm}})-\sum_{m=1}^{M}\left(y_{\mathrm{nm}}\log\left({\bar{y}}_{n}\right)-{\bar{y}}_{n}\right)}$$

Here, *ȳ_n_* is the mean firing of neuron *n* over all *M* time bins, the duration of the recording session. The *pR*^2^ measure is analogous to the variance-accounted-for (VAF) metric, sometimes referred to as R^2^, and commonly used in model fitting with Gaussian statistics. *pR*^2^ is generalized to incorporate the approximate Poisson statistics of the neural spiking data (Cameron and Windmeijer, 1997). In the ratio, the numerator is the difference between the maximum log likelihood achievable by an ideal model in which each prediction *ŷ_nm_* precisely matches the data *y_nm_*, and the log likelihood of the fitted model being evaluated. In the denominator, the first term is again the log likelihood of the ideal model, while the second term is the log likelihood of a model that just predicts the mean firing rate *ȳ_n_*. As each network prediction *ŷ_nm_* approaches its true value *y_nm_*, the pR^2^ value approaches 1. If the deviations from true values are larger than the fluctuations around the mean, then the pR^2^ (like VAF) will be negative. When averaged across all *N* neurons, ${\mathrm{pR}}_{n}^{2}$ provides a principled way of quantifying prediction performance.

## Results

### Intrinsic Measures of Limb State Lead to Better Predictions Than Extrinsic Hand Kinematics

To explore the effect of limb state variables expressed in different coordinate systems, we used as inputs both extrinsic hand kinematics (i.e., the x-y positions, velocities, and accelerations of the hand), as well as intrinsic measures of limb state (muscle lengths, joint angles, and the first derivatives of these measures). In addition, we explored combinations of these inputs. We selected these variables as inputs because of their physiologic relevance and pertinence to previous work.

**FIGURE 1:**
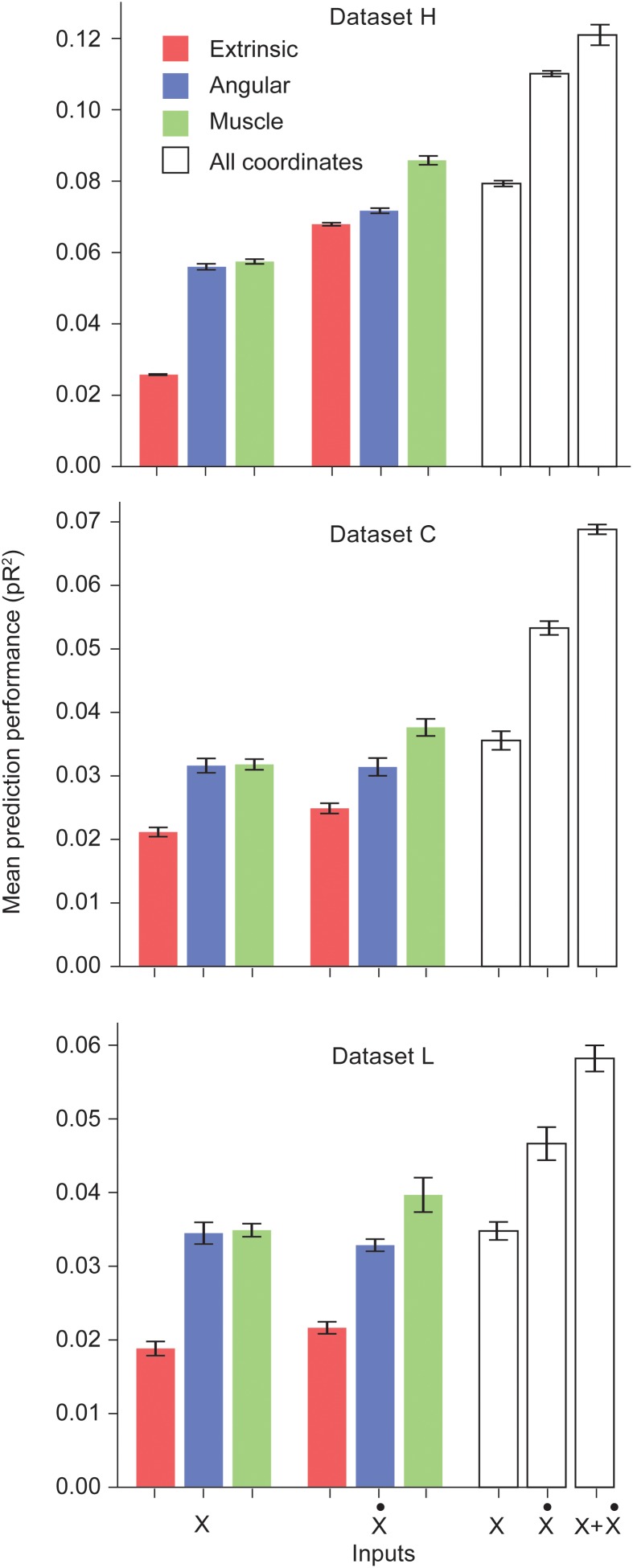
Performance of the ANNs trained on different classes of inputs. The first three bars correspond to networks trained to predict S1 neural activity using different types of kinematic signals as inputs: extrinsic hand coordinates (red), joint angles (blue), and muscle lengths (green). The next three bars correspond to networks trained using the first derivative of these signals. The last three bars indicate encoding performance with all kinematic signals, their derivatives, and the full combination of signals and derivatives, respectively. Each ANN model was trained on the simultaneous prediction of the activity of all recorded neurons.

We trained networks for each of the three datasets. Figure 1 shows the performance of the ANNs trained on a particular type of input for each dataset. Prediction performance was higher when using the intrinsic kinematics of muscle lengths and joint angles, instead of extrinsic hand kinematics. Also, the use of first order derivatives of the various kinematic signals instead of the signals themselves resulted in a further improvement in performance. For all three datasets, we observed a significant increase in performance when using the first derivative of the muscle length signal instead of the signal itself. We also experimented with training ANNs with multiple types of inputs, and observed that using a combination of all extrinsic and intrinsic kinematic signals resulted in an improved performance over separately using each type of signals. Finally, Figure 1 reveals that training with a combination of all signals and their first derivatives leads to a further increase in performance. Further discussion of these results is included in the Discussion section.

**FIGURE 2:**
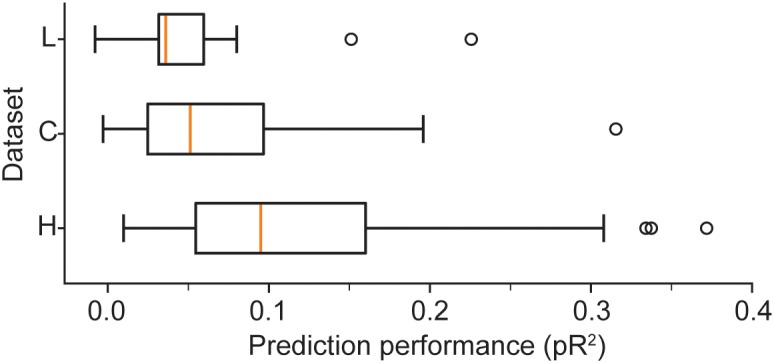
Boxplots illustrate the prediction performance across all neurons for all three datasets: H, L, and C. These results correspond to networks trained with the full combination of signals *X* (hand position, joint angles, and muscle lengths) and their derivatives $\dot{X}$ as inputs.

**FIGURE 3:**
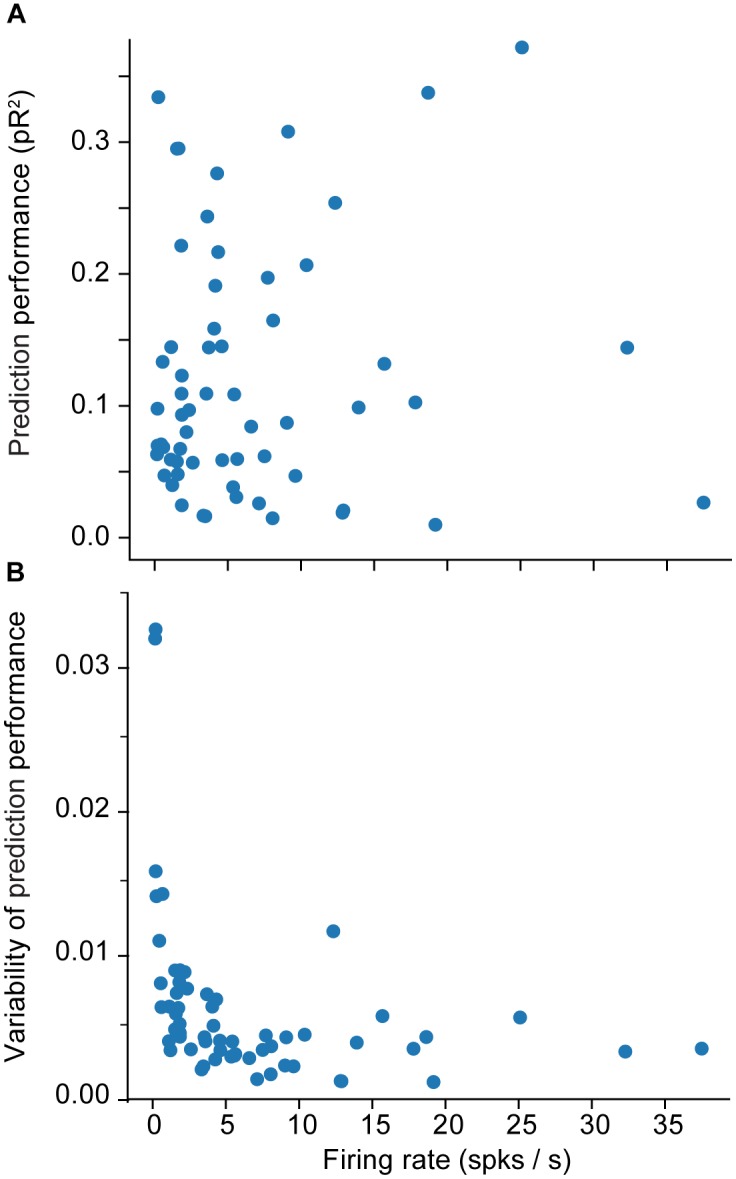
Effect of the firing rate on measures of encoding performance. **(A)** Relation between the firing rate of each neuron and the mean ability to predict its activity as measured by pseudo-R^2^ (*pR*^2^). **(B)** Effect of the firing rate of each neuron on the variability (standard error of the mean; SEM) of prediction performance (*pR*^2^) for that neuron. Here SEM refers to fluctuations across the 10-folds used for cross validation.

We provide a more detailed visualization of ANN performance in Figure 2. For dataset H, in particular, the pR^2^ showed much variability over neurons, ranging from 0.026 to 0.37. A possible explanation for this broad range in performance is the variability in firing rates across neurons. The limited number of data points for neurons with low firing rates may make training less reliable and hinder prediction ability. Alternatively, neurons that fire infrequently might be related to the input signals in a highly nonlinear fashion, in a functional relation that is more difficult for the network to capture. However, there was no correlation between the mean firing rate of a neuron and the mean ability to predict it by using an ANN (measured as pR^2^; Figure 3A).

A more interesting pattern can be seen in the relationship between the mean firing rate of a neuron and the variation in the accuracy of the prediction performance across folds (Figure 3B). Each training experiment for a new fold starts from a new random initial point in the parameter space {*θ*}; the solution could be vastly different across the different folds, particularly when training with insufficient data. Specifically, Figure 3B shows that the lower the mean firing rate, the larger the variability. This indicates that lower firing rates do not provide the ANN with enough data, eventually leading to instability in ANN training.

### Neural Population Covariance Improves Prediction Performance

We expect the ANNs to be able to learn the correlations between neurons, and to use this additional information to make better predictions. To test this, we consider dataset H and instead of fitting all *N* neurons together, we fitted an ANN with two hidden layers on each neuron individually. For each neuron *n*, we compare the performance on that neuron achieved by the network trained to predict all neurons, denoted by ${\mathrm{pR}}_{n,\mathrm{all}}^{2}$, to that achieved by the network trained to predict only the activity of neuron *n*, denoted ${\mathrm{pR}}_{n,\mathrm{ind}}^{2}$. For each neuron *n*, we compute the difference $\Delta {\mathrm{pR}}_{n}^{2}={\mathrm{pR}}_{n,\mathrm{all}}^{2}-{\mathrm{pR}}_{n,\mathrm{ind}}^{2}$.

**FIGURE 4:**
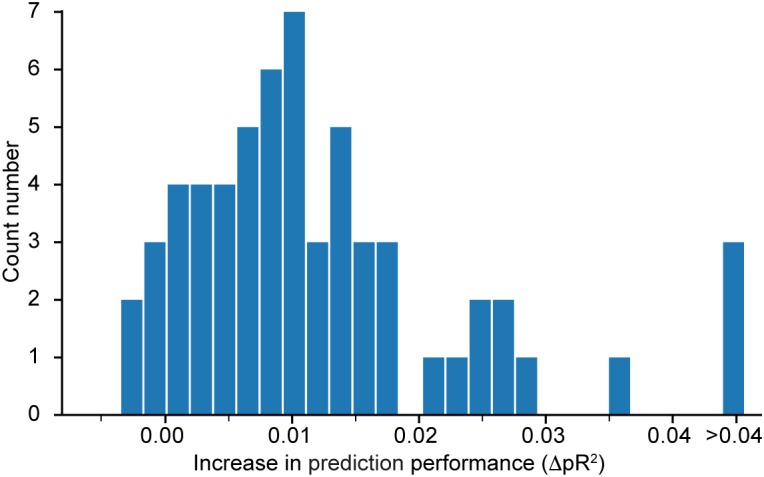
Histogram of $\Delta {\mathrm{pR}}_{n}^{2}$, the difference in pseudo-R^2^ for each neuron in dataset H when subtracting the value obtained from a model that predicts only this specific neuron from the value obtained by the model that predicts all neurons together. A positive value implies an increase in performance in favor of the model trained to predict the activity of all neurons simultaneously.

Figure 4 shows a histogram of the computed differences for all 60 neurons recorded in dataset H. Positive value means that performance increased when the network was trained to predict all neurons simultaneously. Because most of the differences $\Delta {\mathrm{pR}}_{n}^{2}$ are positive, we conclude that the neural network model does indeed benefit from a simultaneous training that incorporates information about correlations in the neural activities. Since the networks were trained using early stopping regularization (see section on the Effect of Regularization on the Predictive Performance of the Model), this effect is not due to potential over-fitting when training networks to predict the activity of single neurons. We restricted this experiment to dataset H because of the high computational demand of calculating $\Delta {\mathrm{pR}}_{n}^{2}$, which requires the additional training of as many networks as the number *N* of recorded neurons in the dataset.

### Recurrent Models Do Not Yield Better Performance

Because our data consists of time sequences, it is natural to ask whether network architectures with recurrence and memory, such as RNNs, would better fit the data by exploiting time correlations. Such architectures may be superior to simple feature engineering approaches where time derivatives are explicitly computed and provided as inputs to feedforward networks.

**FIGURE 5:**
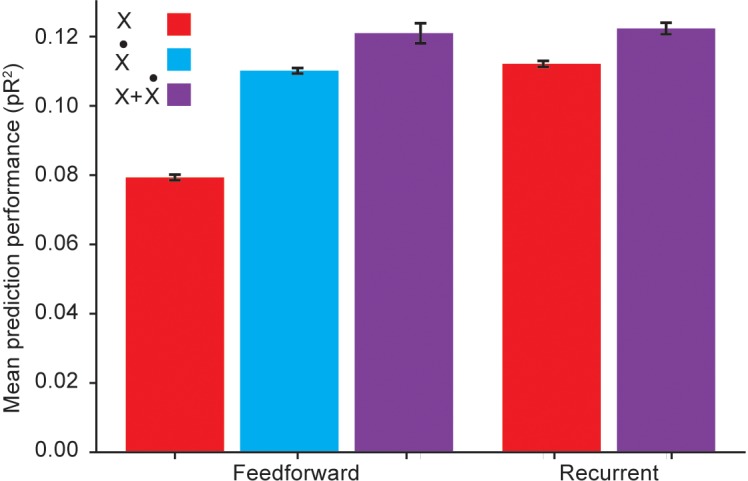
Performance comparison between a recurrent ANN and a feedforward ANN using the same input variables. Here *X* refers to using hand position, joint angles, and muscle lengths as inputs; $\dot{X}$ refers to using the first time derivatives of these signals as inputs; and *X* + $\dot{X}$ refers to using both the signals and their temporal derivatives as inputs.

For these comparisons, we used an RNN with three hidden layers, each composed of 32 units using the hyperbolic tangent as the static nonlinearity. This choice of architecture followed from experimenting with a varying number of hidden layers and units per layer, and choosing the architecture with the best average pR^2^ performance across folds. We investigated the role of temporal correlations by first quantifying the performance of a RNN with inputs given by kinematic signals but not including explicitly calculated temporal derivatives, and comparing it to the performance of a fully connected feedforward ANN with the same inputs. Not surprisingly, the RNN outperformed the feedforward ANN (Figure 5). This implies that the RNN is capable of acquiring internal representations of the temporal derivatives of its input signals to further improve its predictive performance.

However, Figure 5 reveals that if the feedforward ANN is given as inputs the temporal derivatives of the kinematic signals, its performance matches that of the RNN based only on the kinematic signals. We thus conclude that even though the RNN was capable of learning to extract information about temporal derivatives from the original inputs, it did not extract any additional information beyond that available to a simpler feedforward ANN to which these derivatives were explicitly provided as inputs.

We also considered whether RNNs could learn more intricate, higher order temporal information from the temporal differences implicit in the first derivatives. To answer this question, we compared the performance of an RNN to that of a feedforward ANN when both networks were provided with both the kinematic signals and their temporal derivatives as inputs. Figure 5 reveals that in this scenario, RNNs were competitive with fully connected ANNs, but not better. This suggests that the RNN does not extract useful higher order temporal information from these inputs. Because of training difficulties typically associated with RNNs, such as vanishing and exploding gradients (Pascanu et al., 2013), we conclude that feedforward ANNs are a better model for applying deep learning methods to neural encoding problems.

### How Much Data Is Needed to Train an ANNs for Neural Encoding?

**FIGURE 6:**
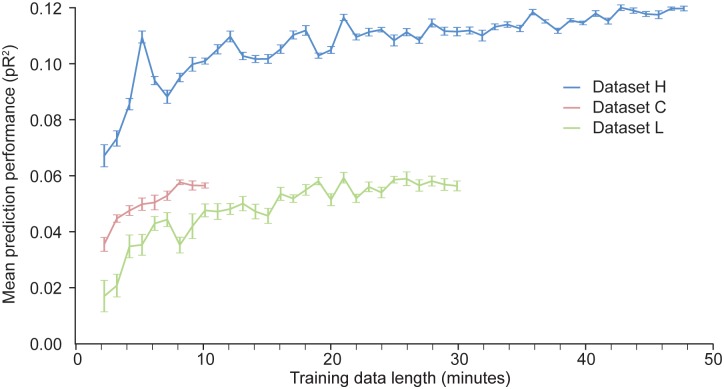
Dependence of the predictive performance of an ANN on the amount of training data. Randomly selected increasingly large contiguous subsets of the datasets H, L, and C were used to train a succession of ANNs.

With artificial neural networks, the amount of available training data is a key factor that determines the network’s performance. If the dataset is too small, the neural network is prone to over-fitting. We have investigated how much data is needed to appropriately fit a two layer ANN trained to predict neural activity in S1. We artificially produced datasets of different sizes by randomly selecting contiguous time windows of different duration within the original training data. We used each of these dataset to train an ANN; the hyper-parameters were kept fixed across these training sessions. The results of these experiments are shown in Figure 6.

As expected, for a given dataset, the more data points are used to train the network, the better the performance. The results for dataset H show that the performance of the ANN eventually reaches an asymptote, beyond which adding more data does not increase performance. Not enough data points were available for dataset C to determine whether the model had reached its asymptotic performance. From the results for datasets H and L, we may conclude that at least 10 min of data are needed to train an ANN to predict S1 neural activity.

### The Effect of Regularization on the Predictive Performance of the Model

In scenarios with short time segments of data available for training, using regularization during ANN training is usually a necessity to prevent over-fitting. Many regularization schemes are available in the neural network learning literature. The most commonly used one is early stopping (Caruana et al., 2001), which uses a separate validation dataset to evaluate the loss function during training. Once the validation loss stops decreasing, training is stopped. All the results reported in previous sections were based on neural networks trained using early stopping, which halts the training procedure when the loss on the validation dataset computed at the end of an epoch is higher than its value computed at the end of the preceding epoch. Another common regularization procedure is weight decay, based on either L1 or L2 norms (Goodfellow et al., 2016). In this approach, an additional term is added to the cost function so as to penalize large values of the parameters {*θ*}. The added term is either λ||*θ*||_1_ or λ||*θ*||_2_, with 0 < λ < 1. One major disadvantage of weight decay over early stopping is that it requires the choice of a value for the hyper-parameter λ, which determines the strength of the regularization.

**FIGURE 7:**
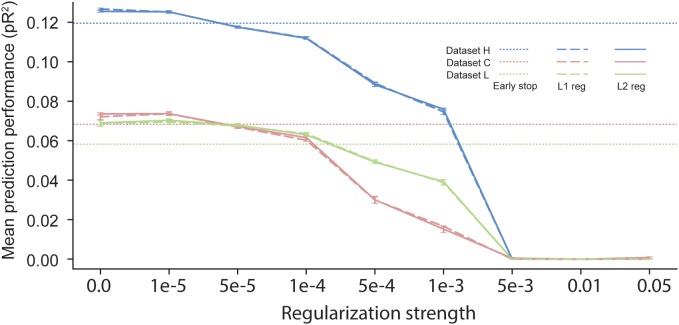
Effects of early stopping, L1, and L2 regularization on the performance of ANN models for the three datasets: H, C, and L. No early stopping was used when either L1 or L2 weight decay regularization was added to the loss function.

We compared the effect of these three different regularization approaches (Figure 7). The performance of networks trained using early-stopping regularization is shown for each of the three datasets by the color-coded horizontal dotted lines. Using weight-decay regularization increased performance only for λ below a threshold that depended on the dataset. Since dataset H included a large number of data points, less regularization was needed to prevent over-fitting, and weight-decay was beneficial only for λ < 10^−5^. In contrast, datasets C and L included a smaller number of data points, and thus they benefited from weight decay with larger values of λ. We noted no significant differences between L1 and L2 weight decay. From these experiments, we conclude that early stopping may be the most reliable choice of regularization for training an ANN to predict neural activity.

## Discussion

The number of studies of the predominantly tactile areas of S1 (areas 3b and 1) greatly exceeds that of proprioceptive areas. A rough indication is that searches for touch and cortex yield more than three times more hits than proprioception and cortex. Of those focused on areas 3a or 2, many have examined movements of the hand or wrist rather than the more proximal limb (Yumiya et al., 1974; Tanji, 1975; Gardner and Costanzo, 1981; Kim et al., 2015), or the grasping portion of a reach (Iwamura and Tanaka, 1996). There is a small number of studies of single-neuron discharge during reaching movements, which conclude that the activity of most neurons in these areas is well correlated with the duration and speed of hand movement (Burbaud et al., 1991) as well as limb posture, often in a nonlinear and spatially nonuniform manner (Tillery et al., 1996). The most comprehensive study of neurons in 3a and 2 found largely linear relations between activity and both movement and endpoint force, as well as a nonlinear, direction-related hysteresis in the tonic, position component (Prud’homme and Kalaska, 1994). More recently, several studies have used both linear and nonlinear methods to decode kinematics from the activity of multiple S1 neurons. Position predictions were either similar to (Weber et al., 2011) or somewhat better (Carmena et al., 2003) than velocity. In another study, linear predictions of hand velocity were more accurate than nonlinear, probably because the latter were overfit (Averbeck et al., 2005).

In recent years there has been great interest in using machine learning techniques, and more specifically layered neural networks (Kietzmann et al., 2019), as models for the prediction of neural activity from parameters of movement (Glaser et al., 2019). This neural “encoding” problem is the complement of the more typical computational problem of “decoding” movement related signals from the activity of many simultaneously recorded neurons. A previous study has compared several different modern machine-learning methods to GLMs in their success to predict the activity of neurons from primary motor and sensory cortex, using hand position, velocity, and acceleration as inputs (Benjamin et al., 2018). Here, we compared the ability of recurrent and feedforward networks to predict the activity of neurons in proprioceptive areas of S1 when using several different types of kinematic signals as inputs. In this approach, we exploit the ability of supervised machine learning methods to identify predictive variables (Glaser et al., 2019).

While recurrent networks can identify and represent temporal dependencies in the data, we noted that simply providing a feedforward network with externally computed, first order temporal derivatives as additional inputs resulted in performance indistinguishable from that of an RNN. For physiologists and neural engineers, this may present an attractive alternative: rather than attempting to construct an RNN model, with all the intricacies of design and training entailed, they may instead provide more pertinent inputs to a feedforward ANN, and attain the same performance. It is the ability of the feedforward ANN to identify predictive variables that circumvents the need for a prior identification of relevant input features.

We also explored the performance benefits of various regularization techniques. Although there was no difference between L1 and L2, there was a range of minimal weight decay for which prediction slightly outperformed that of early stopping. However, this slim advantage is likely outweighed by the decreased performance with only slightly greater weight decay. Furthermore, we monitored the sensitivity of the ANN models to properties of the neural activity (e.g., whether neurons with high firing rates can be better predicted), and investigated whether performance can be enhanced by taking account of the covariance present in the activity of the neural population.

Working with ANNs usually requires access to large datasets, so that optimal fitting to the data does not result in over-fitting and decreased ability to capture the true input-output relation. In many fields, it is possible to synthesize such large datasets artificially. For example, the training sets used for many traditional machine learning tasks such as image recognition or classification can be augmented by surrogate data obtained by applying input transformations known not to modify the output label. This is not possible in applications to neural encoding, as we do not know the nature of variations in sensed input that would not affect the resulting neural activity. It is thus important to know whether the available neural data is sufficient. To answer this question, we investigated the impact of the amount of training data on performance, and found that 10 min of data is adequate for training a two-layer feedforward ANN to predict neural activity in area 2 neurons of S1.

In addition to exploring appropriate network design and training strategies, we can use these results to hypothesize about how neurons in S1 encode information about limb state. Our models indicate that the classic view of neural modulation in area 2, as being determined exclusively by motion of the hand (Prud’homme and Kalaska, 1994), is incomplete. The accuracy of our predictions was increased dramatically by the addition of information about either joint angles or muscle lengths. Furthermore, the activity of these neurons appears to be more directly related to the length and change in length of muscles than to either joint angles or hand position. In addition, we found that the ANNs encoded firing rates more accurately when using the temporal derivatives of the kinematics as their inputs instead of the kinematic signals themselves. These results make good sense given the strong response of muscle spindles to muscle velocity.

Our results suggest that the activity of proprioceptive S1 neurons is most directly a response to low-level information about muscles’ length and change in length. To the extent that the conscious perception of limbs’ state relates to their position and motion in extrinsic coordinates, the additional transformations that map sensory information onto an extrinsic frame would then take place in higher cortical areas (e.g., posterior parietal cortex), presumably in combination with task relevant goals and information from other sensory modalities, particularly vision.

Some of our experimental findings, such as velocity rather than position leading to better performance, were not unexpected. Others were less obvious. For example, the ANNs had lower performance when trained with joint angles instead of muscle lengths, even though the muscle lengths were computed directly from joint angles. In principle, the networks could have learned these relations and acquired internal representations of muscle lengths, however, that was not the case. Similarly, we found that using a combination of extrinsic and intrinsic kinematic inputs led to better performance than either alone. This was also unexpected, as the extrinsic hand position does not provide additional information beyond that already present in muscle length. We may thus conclude that ANNs do not learn to model the relation between intrinsic and extrinsic variables unless explicitly trained to do so.

These results provide a blueprint for the application of machine learning techniques to modeling neural activity in sensory cortices. Although restricted to S1, the implementation issues discussed here should facilitate the application of machine learning techniques to related problems in other areas of the brain. As for S1, our results provide an intriguing hint that this area may be involved in the representation of low-level signals related to muscles’ length and change in length, rather than the classic hand-centric model in extrinsic coordinates (Prud’homme and Kalaska, 1994).

A rapidly emerging area of brain machine interfacing is the development of afferent interfaces intended to restore sensation of the limb to individuals with spinal cord injury or limb amputation (Weber et al., 2012; Tabot et al., 2013; Tyler, 2015; Lebedev, 2016). These afferent interfaces must transform limb state information into stimulus trains applied to the peripheral or central nervous system. The encoding models presented here are well suited to that task.

## Author Contributions

LM and TT designed research. RC collected the data. AL, NR, TT, and RC analyzed the data. AL, TT, NR, SS, AK, and LM wrote the manuscript.

## Conflict of Interest Statement

The authors declare that the research was conducted in the absence of any commercial or financial relationships that could be construed as a potential conflict of interest.

## Supplementary Material

FIGURE S1 Performance comparison between ANNs and a Generalized Linear Model (GLM), essentially an ANN with without hidden layers. A separate ANN or GLM was trained for each neuron in dataset H. We show the comparative performance for those neurons where the GLM pseudo-R^2^ on the test sets was greater than zero. The optimization of the GLM failed for many of the neurons; this suggests that the intrinsic nonlinearities provided by hidden layers are crucial to model the map from inputs to S1 neural activity.

FIGURE S2 Example learning curves for datasets H, C, and L. These learning curves correspond to models trained with the full set of inputs to predict the activity of all recorded S1 neurons simultaneously. Early stopping regularization was used to halt training automatically. The validation set consisted of a random sample of 20% of the training data.
