# Supplementary figures and images for: Neural Networks for Modeling Neural Spiking in S1 Cortex
Source: Front Syst Neurosci. 2019 Mar 29;13:13. doi: 10.3389/fnsys.2019.00013 (PMC6449471; doi:10.3389/fnsys.2019.00013)

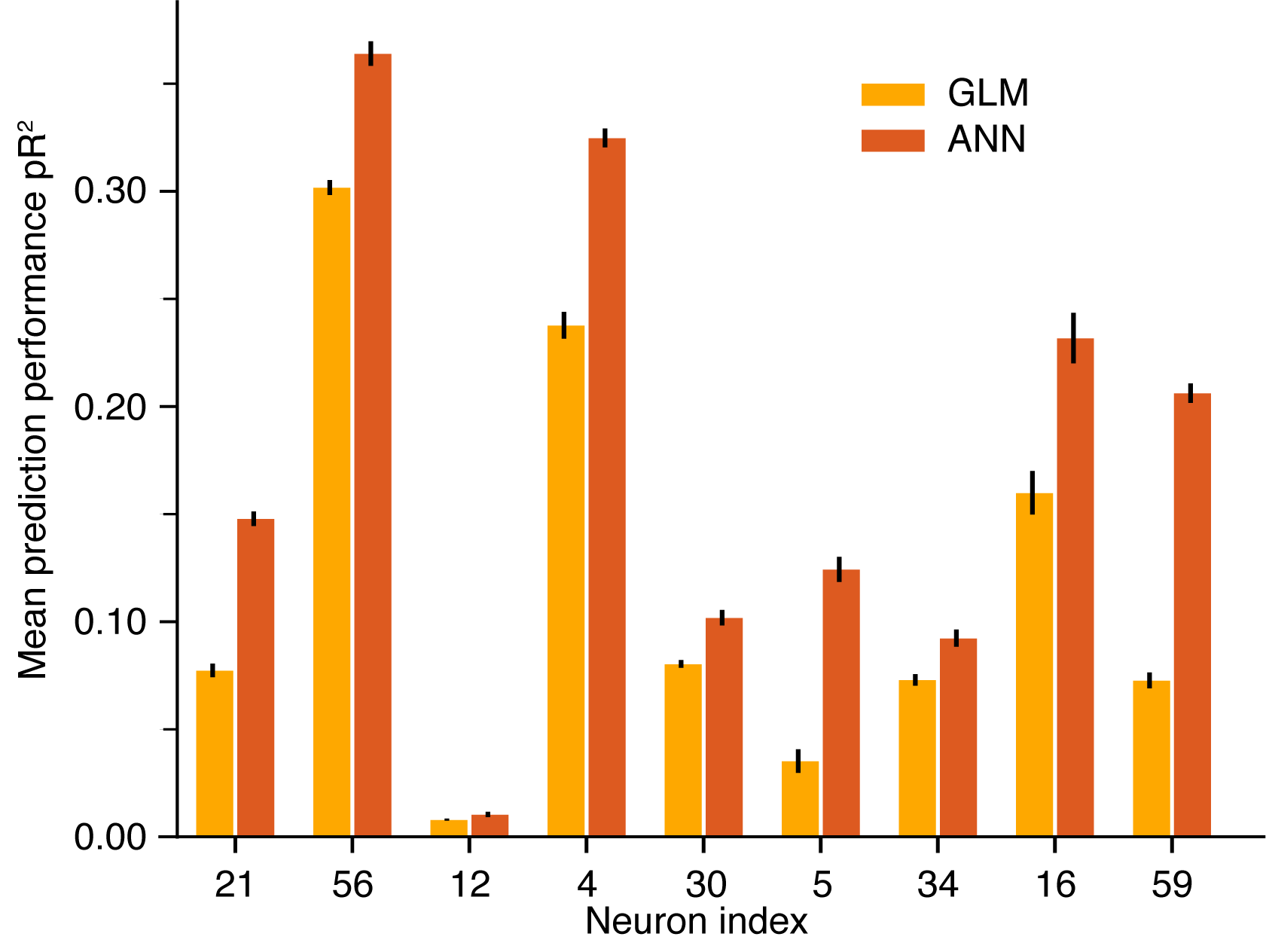

Supplement: FIGURE S1 — Performance comparison between ANNs and a Generalized Linear Model (GLM), essentially an ANN with without hidden layers. A separate ANN or GLM was trained for each neuron in dataset H. We show the comparative performance for those neurons where the GLM pseudo-R2 on the test sets was greater than zero. The optimization of the GLM failed for many of the neurons; this suggests that the intrinsic nonlinearities provided by hidden layers are crucial to model the map from inputs to S1 neural activity. [file Image_1.TIF]

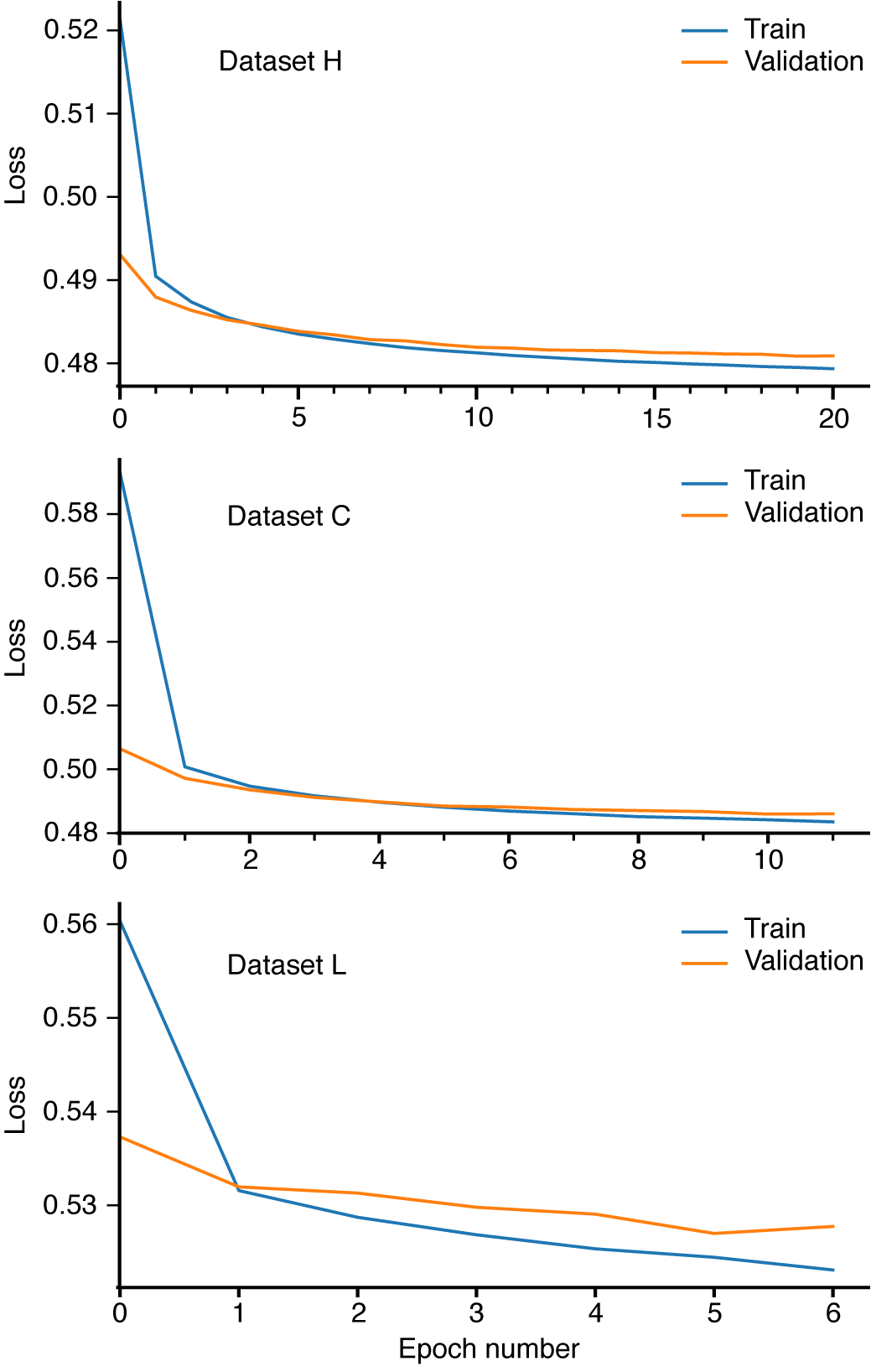

Supplement: FIGURE S2 — Example learning curves for datasets H, C, and L. These learning curves correspond to models trained with the full set of inputs to predict the activity of all recorded S1 neurons simultaneously. Early stopping regularization was used to halt training automatically. The validation set consisted of a random sample of 20% of the training data. [file Image_2.TIF]

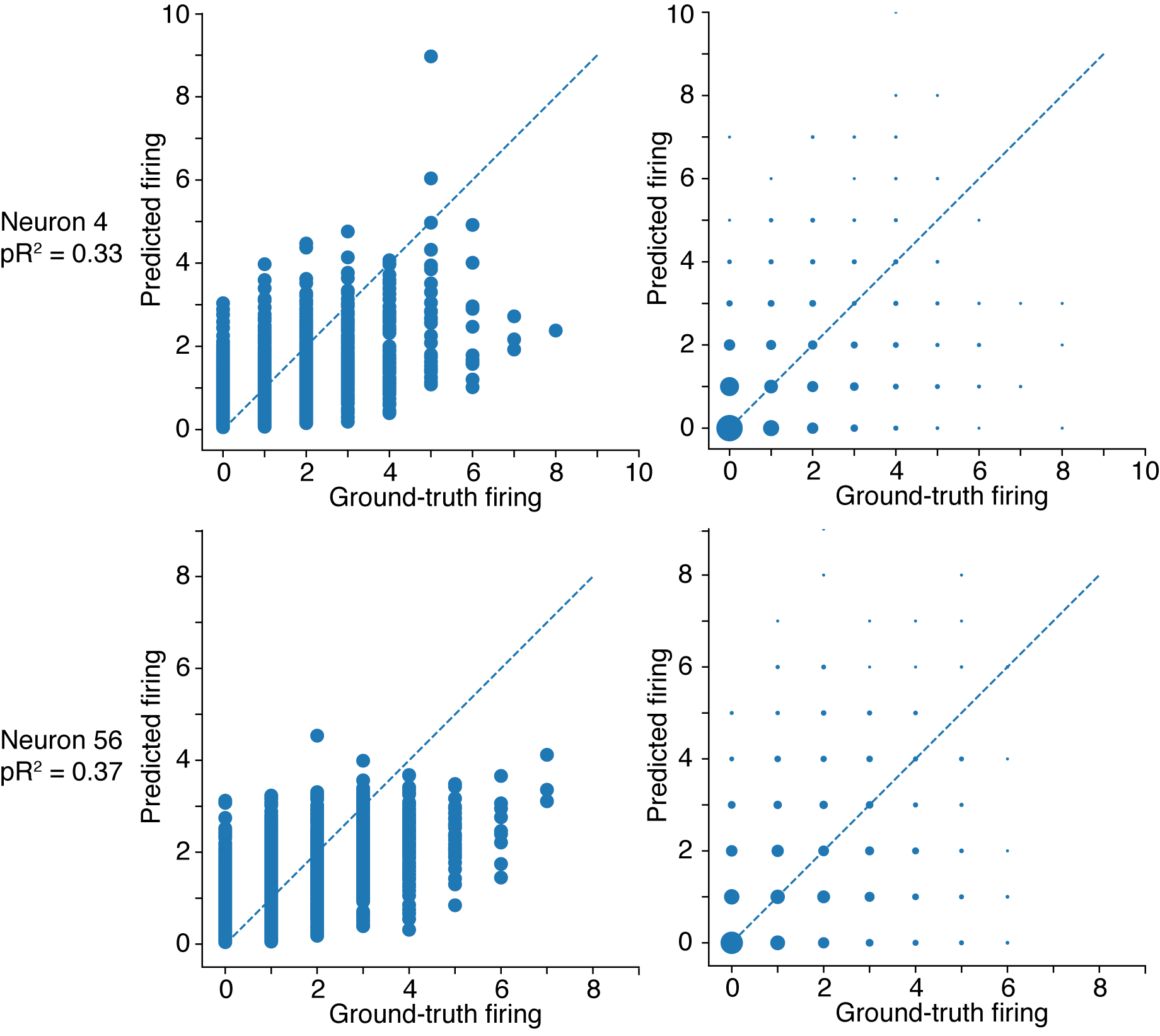

Supplement: Supplementary file 3 [file Image_3.TIF]
